# Long-Term Follow-up of Autologous Fibroblast Transplantation for Facial Contour Deformities, A Non-Randomized Phase IIa Clinical Trial

**DOI:** 10.22074/cellj.2020.6340

**Published:** 2019-09-08

**Authors:** Amir Bajouri, Zahra Orouji, Ehsan Taghiabadi, Abdoreza Nazari, Atefeh Shahbazi, Nasrin Fallah, Parvaneh Mohammadi, Mohammad Rezvani, Zahra Jouyandeh, Fatemeh Vaezirad, Zahra Khalajasadi, Mahshid Ghasemi, Aslan Fanni, Sara Haji Hosseinali, Ahad Alizadeh, Hossein Baharvand, Saeed Shafieyan, Nasser Aghdami

**Affiliations:** 1 Department of Regenerative Medicine, Cell Science Research Center, Royan Institute for Stem Cell Biology and Technology, ACECR, Tehran, Iran; 2 Metabolic Diseases Research Center, Qazvin University of Medical Sciences, Qazvin, Iran

**Keywords:** Cell Therapy, Skin Rejuvenation, Wrinkle

## Abstract

**Objective:**

Recently, the promising potential of fibroblast transplantation has become a novel modality for skin rejuvenation. We investigated the long-term safety and efficacy of autologous fibroblast transplantation for participants with mild to severe facial contour deformities.

**Materials and Methods:**

In this open-label, single-arm phase IIa clinical trial, a total of 57 participants with wrinkles (n=37, 132 treatment sites) or acne scars (n=20, 36 treatment sites) who had an evaluator’s assessment score of at least 2 out 7 (based on a standard photo-guide scoring) received 3 injections of autologous cultured fibroblasts administered at 4-6 week intervals. Efficacy evaluations were performed at 2, 6, 12, and 24 months after the final injection based on evaluator and patient’s assessment scores.

**Results:**

Our study showed a mean improvement of 2 scores in the wrinkle and acne scar treatment sites. At sixth months after transplantation, 90.1% of the wrinkle sites and 86.1% of the acne scar sites showed at least a one grade improvement on evaluator assessments. We also observed at least a 2-grade improvement in 56.1% of the wrinkle sites and 63.9% of the acne scar sites. A total of 70.5% of wrinkle sites and 72.2% of acne scar sites were scored as good or excellent on patient assessments. The efficacy outcomes remained stable up to 24-month. We did not observe any serious adverse events during the study.

**Conclusion:**

These results have shown that autologous fibroblast transplantation could be a promising remodeling modality with long-term corrective ability and minimal adverse events (Registration Number: NCT01115634).

## Introduction

Fibroblasts are the predominant cells of connective tissue that synthetize and organize collagen and other extracellular matrix (ECM) proteins. Furthermore, Fibroblasts secrete soluble cytokines and growth factors such as transforming growth factor-beta (TGF-ß), keratinocyte growth factor (KGF), vascular endothelial growth factor (VEGF), and insulin-like growth factor (IGF) to maintain the structural integrity of the skin (1-3). 

Skin aging is a complex, multifactorial process defined by progressive loss in skin integrity and function (4). The size, amount, and potency of fibroblasts chronologically decline due to natural cellular and molecular events such as reductions in TGF-ß, micro-environment alterations, and Notch signaling disruption (5-8). Aged-fibroblasts secrete higher levels of matrix metalloproteinase that degrade collagen fibrils (9). Since a reciprocal mechanical force between fibroblasts and collagen fibrils is necessary for continuous collagen synthesis, degraded collagen fragments cause a breakdown in the tissue cycle (3, 10, 11). 

On the other hand, destruction of fibroblasts and consequent collagen loss seen in acne scars result from a healing defect after local and systemic inflammation. This defect leads to destruction of the dermal structures with subsequent fibrosis (12, 13). Acne scars occur in 95% of acne participants even during standard treatments, in which 30% progress to significant, permanent scarring with psychosocial complications (14, 15). 

In recent years, the promising potential of autologous fibroblast transplantation has become a novel therapeutic modality for replacement of damaged fibroblasts. In this method, the patient’s retro-auricular area, which has the least damage by UV irradiation, underwent small biopsies to produce the autologous fibroblast cell line through culturing process (16, 17). 

The first autologous fibroblast transplantation was performed in 1995, after which additional studies reported the potentiality of a minimally invasive and autologous rejuvenation method with less complications (16-20). It is presumed that transplanted fibroblasts could stimulate the resident fibroblasts and repair the collagen synthesis system (2, 18, 19, 21). 

Previously, we performed a clinical trial of autologous fibroblast transplantation in 20 participants with wrinkles and acne scars. The results showed its safety and feasibility (unpublished data). In the current study, we aimed to evaluate the long-term safety and efficacy of autologous fibroblast transplantation for participants with mild to severe facial contour deformities. 

## Materials and Methods

### Study design

In this open-label, single-arm, and single center clinical trial, we assessed the efficacy and safety of autologous fibroblast transplantation in wrinkle and acne scar. We estimated the sample size using package long power according to “Sample Size Calculations for Longitudinal Data” by R software (22). In this study, a sample size of 57 participants achieves 80% minimum power to detect a difference using a two-sided binomial test. The powers of other primary hypothesis tests were more than 80%. 

We obtained three punch biopsies from the retro- auricular area in each eligible patient and transferred the biopsy specimens to the Royan Clean Room in order to isolate and cultivate the dermal fibroblasts. After 4 to 5 weeks, cultured fibroblasts were transferred to the clinic and injected into the facial contours of participants over three sessions at 4-6 week intervals. At each treatment session, we injected 0.^6^6 cells) into each cm/cm^2^ of the facial contours. Two independent dermatologists evaluated and recorded the treatment outcome as well as adverse events at 2, 6, 12, and 24 months after the third injection. Participants were also asked to assess their response to treatment at each follow up visit. Furthermore, 5 years after the treatment, we asked the participants by phone to rate the efficacy and durability of the treatment outcome. 

The Institutional Review Board and Ethical Committee of Royan Institute (Tehran, Iran) approved this study. The study was registered at clinicaltrials.gov as NCT01115634.

### Patient selection

Among 76 participants who referred to the Dermatology Clinic of the Royan Institute with wrinkles (49 participants) and acne scars (27 participants) from 2011 to 2018, we recruited 62 participants to the study based on eligibility criteria. We completely explained the treatment process to the participants who met the eligibility criteria. The enrolled participants signed the informed consent before participation to the study. 

### Wrinkle group

We evaluated the participants’ wrinkles based on a 0-7 standard photo guide scoring (20) and included those with wrinkle score of 2-7 (considered as mild to severe). Other eligibility criteria for this group included: 35-65 years of age; wrinkles on the forehead, periorbital, glabella and/ or nasolabial fold (NLF); and total treatment site length of 10-50 cm. 

We did not include participants with history of laser treatment, immune-suppressive therapy, retinoid derivatives, botulinum toxin or temporary fillers within 6 months before recruitment to the study; history of organ transplantation or blood transfusion; any known cancer; known chronic disease; genetic fibroblast or collagen production disorder; permanent or semi-permanent fillers; allergy to animal collagen or its products; sensitivity to local anesthesia; facial plastic surgery or mesotherapy; hepatitis B, hepatitis C or human immunodeficiency viruses (HIV); and pregnant or lactating.

### Acne scar group

We evaluated the participants’ acne scars based on a 0-7 standard photo guide scoring (20) and included those with acne scar score of 2-7 (considered as mild to severe). Other inclusion criteria included: 18-65 years of age; acne scars on the cheek, forehead and/or temporal areas; and total treatment site surface area of 10-50 cm^2^. Exclusion criteria were the same as the wrinkle group. 

### Efficacy profile

Efficacy outcomes were based on comparisons of the baseline and follow-up evaluator and participants’assessment scores. The evaluators and participants rated each wrinkle or acne scar treatment site at the first visit and during follow- up visits, independent of previous scores. We photographed the treatment sites during pre- and post-treatment visits. Two independent, trained dermatologists performed the assessments based on the standard photo guide scoring. We recorded the assessment scores according to the following endpoints: "Responders" were defined as number of treatment sites that had at least a 2 grade improvement compared to the baseline score according to the evaluators’ assessments. Furthermore, we defined the severity of facial contours as mild (grades 2 and 3), moderate (grades 4 and 5), or severe (grades 6 and 7) and then measured the "Responders" of each group in wrinkle and acne scar sites. Participants scored their treatment sites as: -2 (much worse), -1 (worse), 0 (no difference), +1 (better or good), and +2 (much better or excellent) during follow-up visits compared to baseline. We considered the 6-month follow-up evaluations as primary endpoint based on evaluators assessment scores. 

### Adverse events

We prepared a list of probable adverse events before transplantation based on 2010 Common Terminology Criteria for Adverse Events [CTCAE; (23)]. These criteria included local events such as bruising, redness, allergic reaction, pruritus, hemorrhage, nodules and tumors, or systemic events such as infections or allergic reactions. We separately recorded adverse events as well as their duration, severity, and treatment plan of action during the intervention and follow-up visits. 

### Sampling and injection technique

The left retro-auricular area was cleaned with isopropyl alcohol, followed by administration of 2% xylocaine as a local anesthetic. Then, we obtained 3 full-thickness 4-mm punch skin biopsies and transferred the specimens to the Royan Clean Room. We sutured the biopsy sites and then covered them with a sterile dressing.

After isolation and cultivation, the vials that contained cultured fibroblasts were transferred to the clinic. We gently suspended the vial contents and drew it into 1 ml syringes. Before injection, we performed regional blocks via injections of anesthetic agent. The treatment sites were cleaned by an antiseptic solution. Then, the dermatologist injected 0.1 ml of the cell suspension into the superficial and middle layers of dermis of each cm of the wrinkle sites or cm^2^ of the acne scar sites applying a 30-gauge needle. Blanching and wheal formation of the injection site were considered as correct injection of the solution. We did not perform any manipulation on the recipient sites after injection. Participants were avoided the use of chemical soaps or materials to the face for 72 hours after injections. We allowed a short period of indirect application of ice on the treatment sites in the case of long lasting reaction, redness or pain. 

### Cell preparation

The specimens were transferred in 5 ml transporting medium that included Hanks’ balanced salt solution (HBSS, Gibco, Germany) and 1% penicillin/streptomycin (pen/strep, Gibco, Germany) to the clean room. Then, we soaked the skin specimen in 70% ethanol for 30 seconds to reduce contamination, washed with HBSS/pen/strep twice and cut into 2×2 mm pieces using a surgical scalpel blade. We incubated the skin pieces with 1.2 U/ml dispase II solution (Gibco, Germany) for 15-18 hours at 4°C and then 0.1% collagenase type I (Sigma, Germany) for 4 hours at 37°C. We used a Pasteur pipette to pipette the dermis layer in order to release the cells from this layer. The isolated cells were cultured in advanced Dulbecco’s Modified Eagle Medium: Nutrient Mixture F-12 (DMEM/ F12, Gibco, Germany) with 10% fetal bovine serum (FBS, PAA, Austria), 2 mM L-glutamine (Gibco, Germany), and 1% pen/strep. Subsequently, we incubated the cells at 37°C in 5% CO2. We changed the cultured medium every three days. After 4-5 weeks, we collected passage-3 cultured cells, which consisted of 95 ± 17×106 cells for the wrinkle group and 104 ± 15×106 cells for the acne scar group. Next, we divided the cells into three equal parts. We injected the first fresh part of the cells into the treatment sites. The two remaining aliquots were frozen for the later injections. The freezing medium contains 40% DMEM/F12 (Gibco, Germany) 50% (v/v) FBS and 10% (v/v) dimethyl sulfoxide (DMSO). The amount of the injection volume was calculated as: “0.1 × total length or surface of treatment sites”, in which each ml of injection solution contained 5-15×10^6^ cells. Before transplantation, we assessed the cells for any microbial contamination according to sterility, mycoplasma, and endotoxin tests. 

### Immunofluorescence staining

We fixed the cultured fibroblasts with 4% freshly buffered paraformaldehyde, washed with phosphate buffered saline without Ca^2+^ and Mg^2+^ (PBS), and incubated with 10% goat serum, followed by incubation with primary antibody mouse anti-vimentin (Millipore, MAB1687, 1:100) and anti-collagen type 1 (Abcam, ab90395, 1:50). Then, we washed the cells with PBS-and incubated with Donkey anti mouse Alexa 546 (Invitrogen, USA), and anti-mouse IgG (Sigma, USA) for 60 minutes at room temperature. Nuclei were counter-stained with 5 µg/ml of 4’, 6-diamidino-2-phenylindole (DAPI) and analyzed by fluorescent microscopy (Nikon, Japan). 

### Karyotyping

After the cells reached 70% confluence, we added KaryoMAX® Colcemid™ Solution in PBS-10 µg/ml (Gibco, USA) to each flask to a final dilution of 25 µl/ml, which was then incubated at 37°C for 45 minutes. We monitored the changes in cell morphology with an inverted microscope until the fibroblasts detached. For hypotonic treatment, we slowly and carefully added 13 ml of 0.056% KCl (Merck, Germany) with distilled water, followed by incubation at 37°C for 11 minutes, and fixed with methanol: acetic acid (3:1) solution. In order to obtain G-bands, we aged the slides at 60°C overnight. We carried out the staining procedure using Giemsa solution 1:10 (Gibco, USA). Whenever possible, we analysed 15 metaphases. Before printing out each karyotype and counting each chromosome by writing a number on each sister chromatid pair, we observed the slides under a light microscope at ×10 and ×100 magnifications. 

### Statistical analysis

We evaluated the normal distribution of the variables by the Kolmogorov-Smirnov test. We analyzed the normal continuous and non-normal variables with the paired t, Mann-Whitney U, and Kruskal-Wallis tests. We utilized the Spearman and Pearson correlation coefficients to analyze the correlation between variables. We performed the repeated measurement model for groups with related dependent variables that represented different measurements of the same attribute. Values have been expressed as mean ± SD. The level of statistical significance was set at 0.05. We performed the statistical analysis using SPSS version 20 software (SPSS Inc., Chicago, IL, USA).

## Results

### Participants

#### Wrinkle group

**Fig.1:**
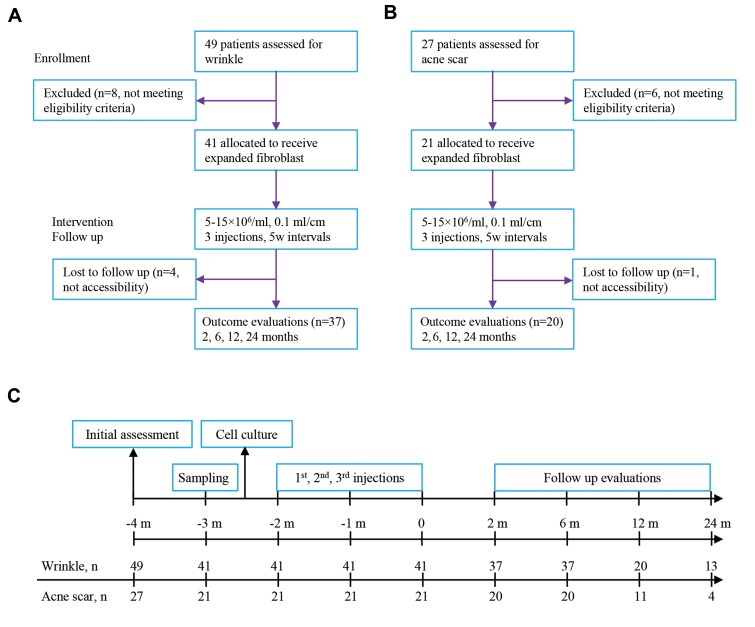
Study design and timeline. A. Study design for wrinkle participants, B. Study design for acne scar participants, and C. Study events and timeline. Eligible participants underwent three autologous cultured fibroblast injections. Efficacy data are based on comparisons of the baseline and follow-up evaluator and patient’s assessment scores.

A total of 49 subjects with contour deformities reffered to the clinic. Table 1 shows the subjects’ baseline characteristics. Eight participants were excluded from the study because of the eligibility criteria or refusal to participate. The remaining 41 participants received autologous cultured fibroblasts. During the follow-up period, 4 participants could not attend the 2- and 6-month follow-up visits and were considered lost to follow-up. Therefore, we analyzed data of 37 participants who had at least 2- and 6-month follow-up visits. We followed 20 participants for 12 months, and 13 participants for 24 months after treatment (Fig .1, Table 1). 

Participants had a mean age of 47 ± 7 years. There were 33 (89.1%) females. Among 132 treatment sites, there were 43 mild, 43 moderate, and 46 severe wrinkles. The average length of the treatment sites was 40 ± 7 cm (range: 11-47 cm). We transplanted an average of 95 ± 17×10^6^ cells during 3 sessions. The mean number of transplanted cells into each treatment site was 0.8 ± 0.3×10^6^ cells/cm. 

#### Acne scar group 

There were 27 subjects who referred to the clinic fromwhich 6 participants either did not meet the inclusion criteriaor declined to participate. A total of 21 participants receivedthe study treatment. One participant was lost to follow-up. We analyzed the data from 20 participants who had at least 2and 6 months of follow-up. There were 11 participants seen atthe 12-month follow-up and we followed 4 participants until 24 months after treatment (Fig .1, Table 1). 

Participants had a mean age of 32 ± 9 years. There were 15 (75%) female participants. Among 36 acne scar sites, there were 5 mild, 16 moderate, and 15 severe acne scars. We transplanted an average of 104 ± 15×10^6^ cells into the treatment sites. The sites had an average surface area of 31 ± 6 cm^2^ (20-44 cm^2^). The mean number of transplanted cells into each treatment site was 1.1 ± 0.3×10^6^ cells/cm^2^. 

### Efficacy outcomes

#### Wrinkle group

The median baseline grade in wrinkle sites was 5 thatdecreased to 3 at 6 months after treatment, based on evaluator’s assessments. Moreover, we observed that at 12 and 24 months following transplantation, this score decreasedto 2. At 2-month follow-up, average response rates of thetreatment sites in comparison with baseline grades were: glabella 1.7 ± 1.3, periorbital 1.5 ± 1.1, NLF 1.4 ± 0.9, andforehead 1.5 ± 1 (P<0.001). At 6 months after transplantation, the response rates were: glabella 2 ± 1.5, periorbital 2 ± 1.3, NLF 2 ± 1.2, and forehead 1.7 ± 1.1 (P<0.001). The mean response rates for all 132 wrinkle sites at the 2- and 6- monthfollow-up visits were 1.5 ± 1.1 and 2 ± 1.2, respectively. 

We observed that at 6 months following transplantation, the responder sites included: 18 (51.4%) for the glabella, 21 (60%) for the periorbital, 18 (64.3%) for the NLF, and 18 (52.9%) for the forehead. Among 132 wrinkle sites, 75 (56.8%) sites were responders. Our assessments 6 months after transplantation showed that 120 (90.1%) sites of the 132 wrinkle sites improved at least one grade. 

**Fig.2:**
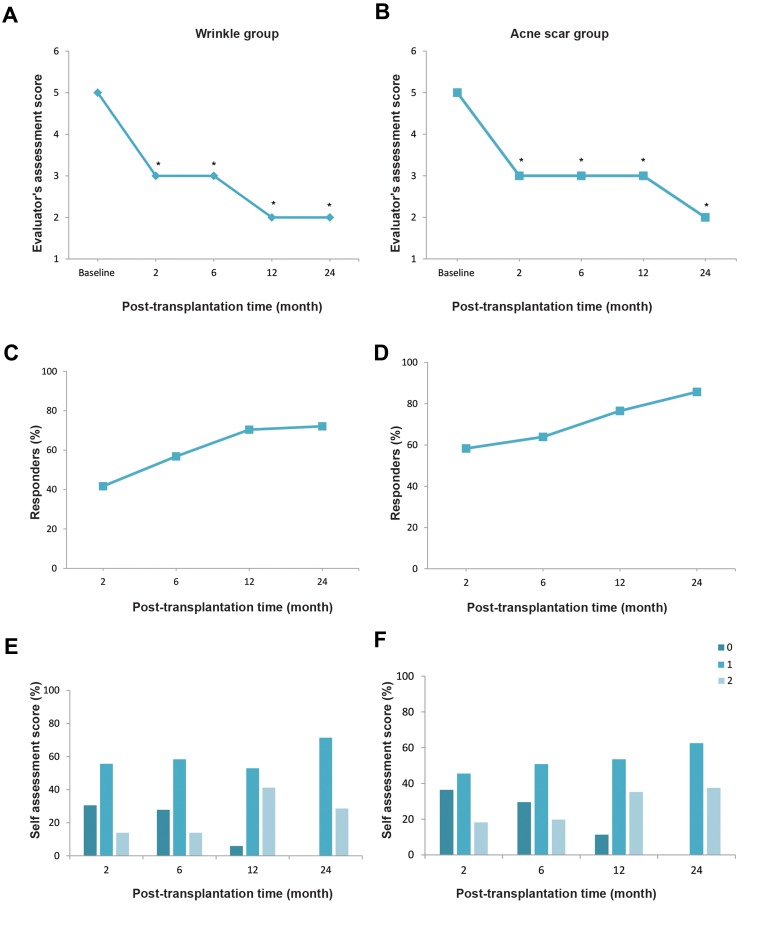
Efficacy outcomes. A. Evaluator’s assessment score of the total sites in participants with wrinkles. Numbers are median, B. Evaluator’s assessment score of the total sites in participants with acne scars. Numbers are median, C. The percentages of participants with wrinkles with a 2-point improvement based on the evaluator assessment, D. The percentages of participants with acne scars with a 2-point improvement based on the evaluator assessment, E. Participants’ self-assessment scores of the total sites in participants with wrinkles, and F. Participants’ self-assessment scores of the total sites in participants with acne scars. *; P<0.05. 0; Nodifference, 1; Better or good, and 2; Much better or excellent.

Participants did not rate any of the treatment sites as -2(much worse) or -1 (worse) after 2 and 6 months of follow- up. Self-assessment scores of +1 (good) or +2 (excellent) were reported at the 2- and 6- month follow-up visits asfollows: 68.5% and 77.1% (glabella), 67.6% and 71.4% (periorbital), 67.8% and 71.4% (NLF), 52.9% and 61.8% (forehead), and 63.6% and 70.5% for all of the 132 wrinkletreatment sites. Table 2 and Figure 2 show the efficacyoutcomes. Furthermore, participants met the 12- and 24month follow-up visits showed sustained efficacy based onevaluator and self-assessment scores (Figes. 2, 3). At 5-yearfollow up, 22 participants were accessible through telephonecontact. The participants scored the treatment sites as+1 (good) or +2 (excellent) in 64.3% of glabella, 75% ofperiorbital, 83.3% of NLF, 42.8% of forehead, and 65.2% of the total 69 wrinkle treatment sites.

**Table 1:** Baseline characteristics of the subjects


Characteristics		Wrinkle group	Acne scar group
		n=37	n=20

Age (Y) (range)		47 ± 7 (35-62)	32 ± 9 (18-45)
Female		33 (89.1)	15 (75)
Sun protection		30 (81.1)	8 (40)
Smoking		8 (21.6)	1 (5)
Previous intervention			
	Laser	10 (27)	12 (60)
	Botulinum toxin	19 (51.3)	-
	Filler injection	7 (18.9)	0
	Microderm	0	10 (50)
	No intervention	12 (32.4)	4 (20)
Treatment sites^*^			
	Glabella	35 (4.1 ± 1.8, 4)	-
	Periorbital	35 (4.9 ± 1.9, 5)	-
	NLF	28 (5.2 ± 1.4, 5)	-
	Forehead	34 (4.4 ± 1.8, 4)	9 (4.3 ± 1.1, 5)
	Temporal	-	7 (5.0 ± 1.6, 5)
	Cheek	-	20 (5.7 ± 1.1, 6)
	Total sites	132 (4.6 ± 1.7, 5)	36 (5.2 ± 1.2, 5)


Data are presented mean ± SD or n (%). *; n (baseline grade; mean ± SD, median) and NLF; Nasolabial fold.

**Table 2:** Six-month follow-up evaluation of subjects


Assessment	Wrinkle group					Acne scar group			
	n=37					n=20			
	Glabella	Periorbital	NLF	Forehead	Total site	Forehead	Temporal	Cheek	Total sites
	n=35	n=35	n=28	n=34	n=132	n=9	n=7	n=20	n=36

Evaluator’s assessment score, median (range)	2 (0-6)	2 (1-6)	3 (1-6)	3 (1-6)	3 (0-6)	3 (2-5)	2 (1-7)	3 (1-7)	3 (1-7)
Responders^a^(%)	18 (51.4)	21 (60)	18 (64.3)	18 (52.9)	75 (56.8)	4 (44.4)	4 (57.1)	15 (75)	23 (63.9)
≥1 grade improvement (%)	32 (91.4)	32 (91.4)	25 (89.3)	31 (91.2)	120 (90.1)	7 (77.8)	6 (85.7)	18 (90)	31 (86.1)
Self-assessment score of +1 or +2 (%)^b^	27 (77.1)	25 (71.4)	20 (71.4)	21 (61.8)	93 (70.5)	5 (55.5)	6 (85.7)	15 (75)	26 (72.2)


^a^; At least 2-grade improvement by evaluator’s assessment, b; Impression of good or excellent by self-assessment, and NLF; Nasolabial fold.

We observed that at 6 months following transplantation, the responder sites of the participants who were less and more than 45 years old were: glabella (28.5 and 66.6%), periorbital (42.8 and 71.4%), NLF (33.3 and 78.9%), and forehead (38.4 and 61.9%), respectively. Additionally, 2 grade improvements were seen in 60.5 and 86.9% of moderate and severe versus 20.9% of mild wrinkle sites (P<0.05).

Participants which had a history of botulinum toxin in forehead and glabella sites showed non-significant better results after fibroblast transplantation compared to the participants without history of botulinum toxin injection(P=0.16 and P=0.19). Also, participants which had a historyof laser therapy on NLF sites showed a mean response rateof 2.5 ± 1.1 versus 1.7 ± 1.2 in participants without history of laser therapy (P=0.12). Participants used to smoke did notshow significant difference on response rates compared to non-smoker participants (P=0.98). 

**Fig.3:**
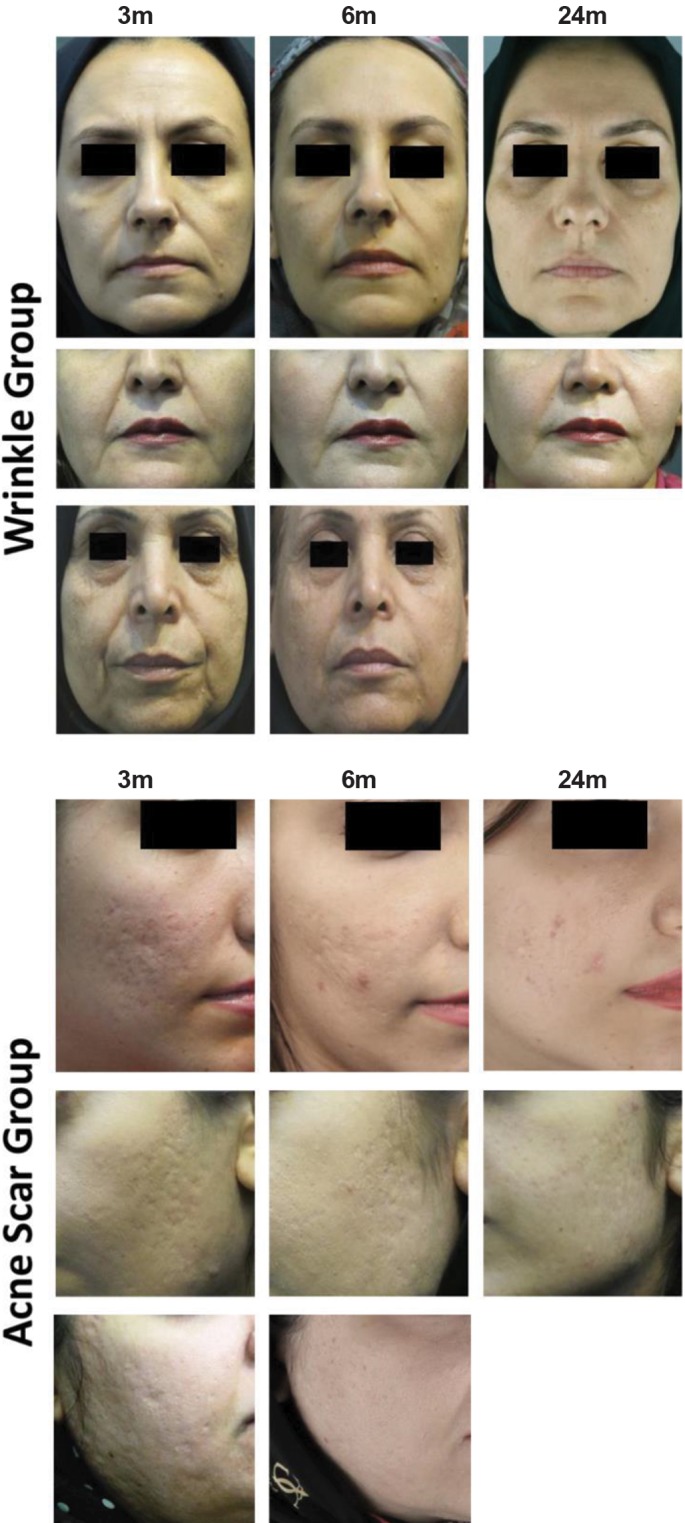
Participants underwent autologous cultured fibroblast transplanta- tion before and after treatment.

#### Acne scar group

The median baseline grade in acne scar sites was 5 that decreased to 3 at 6 months after treatment, based on evaluator’s assessments. Moreover, we observed that at 24 month following transplantation, this score decreased to 2. Average response rates of treatment sites in comparison with baseline grades at the 2-month follow-up were: 2.1 ± 1.1 (cheek), 1.9 ± 1.3 (temporal), 1.1 ± 0.9 (forehead), and 1.8 ± 1.3 for all 36 acne scar sites (P<0.001). The 6-month response rates were: 2.2 ± 1.2 (cheek), 2 ± 1.4 (temporal), 1.4 ± 1.1 (forehead), and 2 ± 1.2 for all 36 acne scar sites (P<0.001, Figes.2, 3). 

The evaluators noted that 15 (75%) sites in the cheek, 4 (57.1%) in the temporal area, 4 (44.4%) from the forehead, and 23 (63.9%) out of the total 36 acne scar sites were responders at 6 months follow-up. However, 31 (86.1%) of all acne scar sites had at least a one grade improvement. 

Participants scored +1 or +2 (good or excellent) for the following sites at the 2- and 6- month follow-up visits: forehead (55.5 and 55.5%), cheek (75 and 75%), and temporal (71.4 and 85.7%). At 2- and 6- month after final transplantation, a total of 69.4 and 72.2% of all 36 acne scar sites scored +1 or +2, respectively (Fig .2). As seen in Table 2, participants did not rate any of the treatment sites as -2 (much worse) or -1 (worse). Furthermore, participants met the 12and 24- month follow-up visits showed sustained efficacy based on evaluator and self-assessment scores (Figes.2, 3). At 5-year follow up, 10 participants were accessible and 30% of the participants declared the durability of the treatment on the acne scar sites.

At 6 month following transplantation, 2 grade improvements were seen in 68.7 and 73.3% of moderate and severe versus 20% of mild wrinkle sites (P<0.05). Additionally, participants which had a history of laser therapy or microderm abrasion on cheek site showed a similar response rate compared to the participants without such history (P=1). 

#### Adverse events

All participants experienced temporary, mild burning during and 1-2 hours after transplantation. A total of 3 participants with acne scars and 8 with wrinkles complained of mild to moderate adverse events that included bruising and redness. Of these, 9 reported that the adverse events spontaneously resolved after 24-48 hours. However, bruising in 2 scar participants lasted for 4-5 days which resolved following the application of ice and oral NSAID administration. We observed no major or systemic adverse events during the 24 months of follow- up evaluations. 

### Cell characteristics 

**Fig.4:**
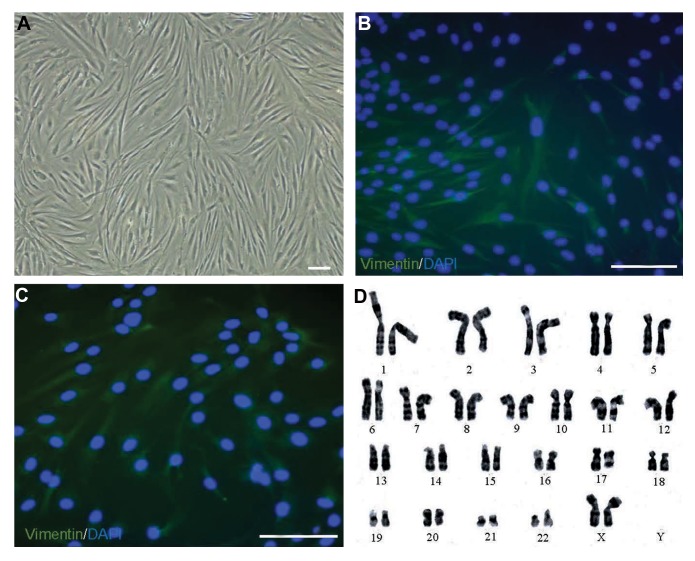
Characteristics of cultured fibroblasts. A. Phase-contrast microscopy of fibroblasts shows spindle and elongated cells after cultivation (scale bar: 50 µm). Representative fluorescent staining shows: B. Vimentin (scale bar: 100 µm), C. Collagen type I expression in cultured fibroblast cells (scale bar: 100 µm). Nuclear stained by DAPI (blue), and D. Karyogram of cultured fibroblasts indicates no abnormality in third passage.

After 4-5 weeks, we collected passage-3 fibroblasts. The fibroblasts showed a spindle-shaped morphology in the culture (Fig .4A). Our data indicated that cell viability at the first transplantation was 97.8 ± 3.5%, whereas the second injection had cell viability of 92.8 ± 12.2% and 93.3 ± 10.1% for the third injection. Immunostaining of cultured fibroblasts showed high-level expressions of vimentin and collagen type 1 (Fig .4B, C). We assessed karyotypes of passage-3 fibroblasts for genomic stability. There were normal 46,XX and 46,XY karyotypes in all participants with no evidence of any abnormality (Fig .4D). 

## Discussion

During the past two decades, many biodegradable and non-biodegradable dermal filler substances such as collagen, hyaluronic acid, and fat grafting have been introduced for reducing facial contour deformities. However, these approaches do not result in durable effects, and are associated with short- and long-term adverse events such as local or systemic infections, injection site abscesses, hypersensivity, nodules, tissue necrosis, and immune reactions. Recently, injection of permanent synthetic fillers for soft tissue augmentation has become common. However, a growing amount of literature has described complications following injection of permanent filling agents such as indurations, infections and inflammations, abscesses, and delayed granulomas (9, 24). 

Recent efforts led to the introduction of autologous fibroblast transplantation as a natural corrective approach with fewer adverse events and longer efficacy. The studies have reported a mean improvement of 2 scores based on clinical scorings and responder rates of 30-82.2% (16, 17, 19, 20). Investigations on safety and efficacy of this modality has led to FDA approval of autologous fibroblast applications for nasolabial wrinkles (25). Our study also showed a mean improvement of 2 scores in the wrinkle and acne scar treatment sites at the 6-month follow up, which remained stable for a mean time of 24 months. Our results have revealed that responder rates of 56.8% for the wrinkle group and 63.9% for the acne scar group. The higher efficacy rate in the acne scar group agreed with similar studies and demonstrated that participants with acne scars experienced greater benefits from fibroblast transplantation (16). This could be attributed to subcisionlike effect of the injection procedure, which is a common treatment for acne scars. 

In the present study, 70.5% of participants with wrinkles and 83.3% of participants with acne scars expressed satisfaction with the clinical results at the 6-month follow up. There was an upward trend observed in the scores reported by participants during the follow up visits, so that all participants assessed the effect of fibroblast transplantation as 'good' or 'excellent' at the 24-month follow up. This prolonged efficacy might be attributed to previous observation of live and bioactive fibroblasts in the recipient area up to 12 months after transplantation (17, 26). However, participants with acne scar reported a decrease in the self-assessment score from 72.2% at 1-year to 30% at 5-year post transplantation, and participants with wrinkle declared a milder decrease in the primary achieved outcome, from 70.5% at 1-year to 65.2% at 5-year post transplantation. 

It is presumed that the efficacy rate of fibroblast transplantation may be affected by several variables, which include treatment location, treatment site severity, patient’s age, and treatment protocol. Here, we have observed a lower response rate in the forehead area of both wrinkle and acne scar groups. A higher response rate was seen in the cheek area of participants with acne scars, whereas we did not detect any remarkable higher response rates in the different wrinkle treatment sites. However, West and Alster (27) previously reported a higher efficacy of fibroblast transplantation in NLFs compared to lip and glabella wrinkles. In this study, we observed that moderate to severe treatment sites showed higher efficacy rate compared to the mild sites. We did not observe any significant correlation between age and final outcome, however, participants aged more than 45 years old showed more responder sites compared to the younger participants. Previously, two studies showed similar culture characteristics between aged and young fibroblasts (17, 28). However, two studies previously demonstrated lower responses in fibroblast transplantation for older participants (18, 29). 

Regards to the treatment dosage, we injected 0.5-1.5×10^6^ fibroblasts per cm or cm^2^ of the treatment sites in each session depends on the sites’ length or area. Previously, Weiss et al. (20) injected 2×10^6^ fibroblasts per cm or cm^2^ of wrinkles or acne scars in each session. Later, the study that evaluated the efficacy of autologous fibroblasts for wrinkles, amounts of 1-2×10^6^ cells/ cm were administered to the treatment sites (19). However, Zorin et al. (17) who evaluated the effects of fibroblast therapy in wrinkle, administered 0.7×10^6^ of cells per cm of treatment sites. Dose finding studies would be necessary to define the optimum cell dose in fibroblast transplantation for contour deformities. 

Previous studies indicated that cultured fibroblasts from passages 5, 10, or higher maintain their genomic stability with no mutations or translocations in the cultivation process. However, greater proliferation capacity and higher secretion bioactivity were observed in fibroblasts of passages 3 or 4 (30, 31). Therefore, we injected passage-3 fibroblasts and evaluated the cells according to biosynthetic activity and karyotype normality before transplantation. In our study, we did not observe any serious adverse events over 24 months of follow up, with the exception of temporary, mild reactions that resolved within a few days after transplantation. 

Some limitations to the study presented here need to be declared. First, there is no control group in our trial. Second, our results are based on semi-objective assessments.

## Conclusion

Our study demonstrated that autologous fibroblast transplantation could be a promising remodeling modality, especially for moderate to severe facial contour deformities in terms of long-term corrective ability with no adverse effects. These results are encouraging to conduct large randomized clinical trials to optimize the treatment protocol. 
